# ATP-binding cassette transporter A1 gene polymorphisms and serum lipid levels in young Greek nurses

**DOI:** 10.1186/1476-511X-10-56

**Published:** 2011-04-13

**Authors:** Vana Kolovou, Genovefa Kolovou, Apostolia Marvaki, Agathi Karakosta, Georgios Vasilopoulos, Antonia Kalogiani, Dimitrios Degiannis, Christina Marvaki, Constantinos A Demopoulos

**Affiliations:** 1 Molecular Immunology Laboratory, Onassis Cardiac Surgery Center Athens, Greece; 2 1st Cardiology Department, Onassis Cardiac Surgery Center Athens, Greece; 3 Thriassio General Hospital, Magoula, Attica, Greece; 4 Department of Nursing, A' Technological Educational Institute of Athens, Greece; 5 Department of Chemistry, National and Kapodistrian University of Athens, Greece

## Abstract

**Objective:**

The ATP-binding cassette transporter A1 (ABCA1) is essential protein involved in lipid metabolism. The present study was undertaken to detect the possible association of polymorphisms in the ABCA1 gene [rs2230806 (R219K) and rs2230808 (R1587K)] and lipid profile in Greek young nurses.

**Methods:**

The study population consisted of 308 unrelated nurses who were genotyped and the ABCA1 polymorphisms were detected. Additionally, lipid profile [total cholesterol (TC), triglycerides (TGs), high density lipoprotein cholesterol (HDL-C), low density lipoprotein cholesterol (LDL-C) and apolipoprotein (apo) A] was evaluated.

**Results:**

There was no difference in the genotypic and allelic frequencies of the R219K polymorphism according to lipid profile. The R1587K genotypes differed significantly according to TC, LDL-C and TGs concentration (p = 0.023, p = 0.014 and p = 0.047, respectively). Particularly, significant difference in TC, LDL-C and TGs concentration was detected between RK and RR genotypes (p = 0.006, p = 0.004, p = 0.014, respectively). Women with RK genotype compared to RR genotype had higher concentration of TGs (134.25 mg/dl vs 108.89 mg/dl, p = 0.014, respectively), total cholesterol (207.41 mg/dl vs 187.69 mg/dl, p = 0.006, respectively), and LDL-C (110.6 mg/dl vs 96.9 mg/dl, p = 0.004, respectively).

**Conclusions:**

These findings suggest that the R1587K polymorphism of ABCA1 gene was associated with lipid profile of Greek nurses. Women with RK genotype had higher TGs, total and LDL-C concentration compared to RR genotype. These observations may be significant in assessing the risk of CAD since a 1% change in LDL-C is associated with a 1% change of cardiovascular events. Also, TGs concentration were documented to play a significant role in women. However, this needs to be confirmed by larger studies.

## 1. Introduction

The ATP-binding cassette transporter A1 (ABCA1) acts as a vehicle for cellular cholesterol which after crossing cell membrane bounds to acceptor molecule such as apolipoprotein (apo) A [1-3]. Thus, ABCA1 influences the initial steps in high density lipoprotein (HDL) formation and in reverse cholesterol transport. The ABCA1 protein belongs to ABC proteins family, which are ingredients of biological membranes and use ATP to transfer various particles such as lipids [1]. The ABCA1 protein gene is located in the chromosome 9 in the area 9q31.1. This gene encodes a protein which is expressed in many tissues such as liver, macrophages, intestines, lungs etc. Several ABCA1 gene polymorphisms were identified, including rs2230806 (R219K) and rs2230808 (R1587K), which are mainly associated with the HDL cholesterol (HDL-C) concentration. The R219K results in a single amino acid change in codon 219 from arginine to lysine. The K allele of the R219K polymorphism has been related to low coronary artery disease (CAD) risk [4] and to lower triglycerides (TGs) concentration [5]. As far as concern the levels of HDL-C the reports are still confusing [4,6]. The R1587K which is located in the extracellular loop of the ABCA1 protein, results in a single amino acid change in codon 1587. This polymorphism has been consistently associated with low HDL-C concentration [7,8].

This study was undergone to evaluate the influence of these two ABCA1 gene polymorphisms on lipid profile [total cholesterol, TGs, HDL-C and low density lipoprotein cholesterol (LDL-C)] in young nurses. We also tested if there are any differences in frequency of ABCA1 gene polymorphisms between individuals with low and high HDL-C concentration.

## 2. Materials and methods

### Subjects

The genotyping of 308 Greek female students aged 22.5 (±2.3) years who were attended to the University of Nursing of Technological and Educational Institution was performed. All students had no personal history of CAD and were not taking any drugs. Also, exclusion criteria were diabetes mellitus, thyroid and liver disease, high alcohol consumption, professional athleticism and any chronic disease.

All women were attended to the University every day and were staying for 8-10 hours. Women were eating at the school canteen which served typical Mediterranean food. Only one (evening) meal daily was most likely to be different in each student.

Additionally, subject were divided to those with high (HDL-C >70 mg/dl) and low (HDL-C <40 mg/dl) HDL-C concentration.

The University of Nursing of Technological and Educational Institution ethics committee approved the protocol of this study. All subjects signed an informed consent form.

### Blood Chemistry

Plasma total cholesterol, TGs, HDL-C and apo A1 were measured using enzymatic colorimetric methods on Roche Integra Biochemical analyzer with commercially available kits (Roche). The serum LDL-C concentration was calculated using the Friedewald formula only in patients with TGs concentration < 400 mg/dl.

### DNA analysis and determination of blood lipids

The ABCA1 gene polymorphisms (R219K and R1587K) were detected using polymerase chain reaction (PCR) and restricted fragment length polymorphism analysis (RFLP's). The PCR was performed using Taq polymerase KAPATaq. For R219K polymorphism the oligonucleotide primers which were used are AAAGACTTCAAGGACCCAGCTT and CCTCACATTCCGAAAGCATTA [9]. PCR was subjected to 95°C for 5 min, thirty cycles of 95°C for 30 s, 55°C for 30 s and 72°C for 30s and final extension to 72°C for 7 min, producing a fragment of 309 bp. This fragment was subsequently cleaved by EcoNI, creating fragments for R allele 309 bp and for K allele 184 bp and 125 bp, which were subjected to electrophoresis on an agarose gel 4% and visualized with ethidium bromide.

**Figure 1:**
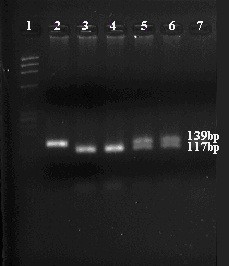
**R1587K gene polymorphism**.

**Figure 2:**
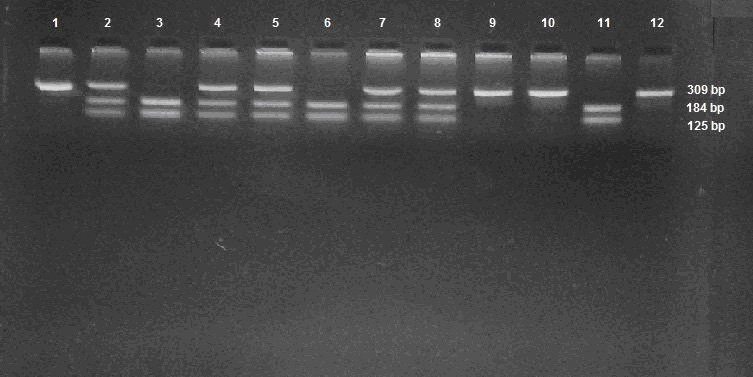
**R219K gene polymorphism**.

For R1587K polymorphism the oligonucleotide primers which were used are AAGATTTATGACAGGACTGGACACGA and TGAATGCCCCTGCCAACTTTAC [8]. PCR was subjected to 95°C for 5 min, thirty cycles of 95°C for 30 s, 60°C for 30 s and 72°C for 30s and final extension to 72°C for 7 min, producing a fragment of 139 bp. This fragment was subsequently cleaved by BssSI, creating fragments for R allele 117 bp and 22 bp and for K allele 139 bp, which were subjected to electrophoresis on an agarose gel 4% and visualized with ethidium bromide (Figure 1, Figure 2).

### Statistical analysis

The results are given as mean ± standard deviation (SD) or as median and interquartile range (IQR) according to normality of continuous variables. All qualitative variables are presented as absolute or relative frequencies. All biochemical variables were assessed for normality of distribution employing the Shapiro-Wilk test and non parametric statistical tests were used if appropriate. However, non parametric variables were initially normalized; TGLs by the log10 transformation - apoA1 and TC by square root transformation and parametric criteria were employed, all providing the same results as non parametric tests. However, TGL variable was extremely skewed and we decided to use the median [IQR] presentation and keep the result of the non parametric test that was employed.

Differences in lipid levels for the various genotypes were evaluated with one - way analysis of variance (ANOVA) or its non-parametric analogue Kruskal - Wallis H statistic. The Pearson's chi-square test was employed for the categorical variables.

All tests were two-tailed and statistical significance was established at 5% (p < 0.05). Data were analyzed using Stata™ (Version 10.1 MP, Stata Corporation, College Station, TX 77845, USA).

## 3. Results

### Clinical and laboratory parameters

Demographic data, clinical characteristics and lipid profile of the study cohort are shown in Table 1. R and K allele frequencies appear to have equal distributions in both ABCA1 polymorphisms (p > 0.05) (Table 2). The frequencies of R219K genotypes were 50.97% for RR, 40.91% for RK and 8.12% for KK, whereas the frequencies of R1587K genotypes were 47.08% for RR, 41.56% for RK and 11.36% for KK. Both frequencies were found in Hardy-Weinberg equilibrium.

**Table 1:** Characteristics of the study population.

*Demographic data*		*Lipid profile(in mg/dl)*	
Number of subjects	308	Total Cholesterol	196.6 (59.7)
Age (ys)	22.5 (2.3)	TGs	87 [60.5 - 149]
BMI (Kg/m^2^)	21.5 [19.8 - 24.2]	HDL-C	69.2 (25.9)
Waist (cm)	87.0 (12.5)	LDL-C	103.5 (38.8)
		Apo A	152.8 (53.5)

***Clinical characteristics***			

Smoking (yes/no)	110/176 (38.5%/61.5%)		

HDL-C: high density lipoprotein cholesterol, LDL-C: low density lipoprotein cholesterol, ApoA1: Apolipoprotein A1, TGs: triglycerides. Data are expressed as mean ± standard deviation (SD) or as median and interquartile range (IQR) according to normality of continuous variables. Qualitative variables are presented as absolute and relative frequencies.

**Table 2:** R and K allele frequencies according to R219K and R1587K polymorphisms.

ABCA1	R allele frequency	K allele frequency	p value*
**R219K**	0.72	0.28	0.11
**R1587K**	0.68	0.32	

Fisher's exact test

### R219K and R1587K polymorphisms

The distribution of R219K and R1587K polymorphisms was investigated according to Low HDL-C (n = 46) and High HDL-C concentration (n = 104). No statistical difference was observed in both polymorphisms when compared to Low vs High HDL-C concentration (p = 0.44 and p = 0.48, respectively). Moreover, no difference in the distribution of the R219K genotypes was detected according to lipid profile (Table 2). Also, no differences in the distribution of K and R carriers of R219K polymorphism was detected according to lipid profile (p = 0.87).

**Figure 3:**
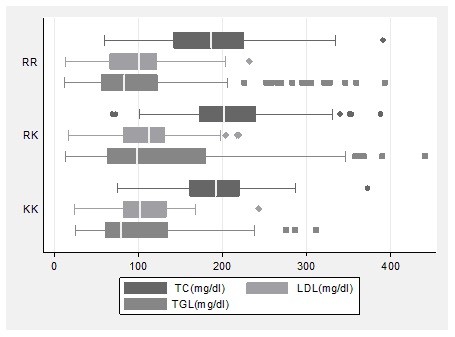
**Box plot of total cholesterol, LDL-C and TGs concentration according to R1587K genotypes**.

The R1587K genotypes differed significantly according to total cholesterol, LDL-C and TGs concentration (p = 0.023, p = 0.014 and p = 0.047, respectively) (Table 3). Significant difference in LDL-C concentration was detected between RK and RR genotypes of the same polymorphism (110.6 mg/dl vs 96.9 mg/dl, respectively, p = 0.004), Figure 3. However, the LDL-C concentration did not differ between women with the KK and RK genotype of the R1587K polymorphism (104.52 mg/dl vs 110.64 mg/dl, p = 0.4). Total cholesterol levels was higher in women with the RK compared to women with the RR genotype of the R1587K polymorphism (207.41 mg/dl vs 187.69 mg/dl, p = 0.006), whereas total cholesterol levels did not differ between women with RK and KK genotypes (207.41 mg/dl vs 193.66 mg/dl, p = 0.22). Finally, a significant difference was observed in the levels of TGs according to the R1587K polymorphism, with the RK genotype women having higher TGs concentration in comparison to the RR genotype (134.25 mg/dl vs 108.89 mg/dl, p= 0.014). However, TGs levels did not seem to differ between RK and KK genotypes of the same polymorphism (134.25 mg/dl vs 108.06 mg/dl, p= 0.11). Also, no differences in the distribution of K and R carriers of R1587K polymorphism was detected according to lipid profile (p = 0.5).

**Table 3:** Blood lipid levels according to ABCA1 R1587K polymorphism in all genotypes.

*Lipid Profile (in mg/dl)*	Genotype	Mean	SD	P*
	RR	187.69	59.19	
***Total cholesterol***	RK	207.41	59.55	0.023
	KK	193.65	57.49	
	RR	68.94	26.35	
***HDL-C***	RK	69.89	25.33	0.88
	KK	67.48	26.57	
	RR	96.97	38.48	
***LDL-C***	RK	110.64	37.26	0.014
	KK	104.52	41.51	
	RR	147.8	53.23	
***ApoA1 (mg/dl)***	RK	158.46	54.33	0.26
	KK	152.54	50.65	

	**Genotype**	**Median**	**IQR**	**P**^*†*^

	RR	83	[56 - 123]	
***TGs***	RK	98	[63.5 - 180]	0.047
	KK	79	[61 - 134]	

HDL-C: high density lipoprotein cholesterol, LDL-C: low density lipoprotein cholesterol, ApoA1: Apolipoprotein A1, TGs: triglycerides. *P values among genotypes from Anova test performance - ^†^P values among genotypes from Kruskal Wallis test performance.

## 4. Discussion

We examined the probable impact of the ABCA1 polymorphisms as a genetic influence on lipid profile in Greek young nurses.

### R219K gene polymorphism

The frequency of R allele of R219K polymorphism in our study was 72% similar to Pasdar et al [10] who reported 73.2% in controls (68% in patients with ischemic stroke) and Porchay et al [11] [D.E.S.I.R participants (Data from an Epidemiological Study on the Insulin Resistance)] who reported 71.7%. Conversely, Clee et al [5] reported frequency of 46% in Dutch men with proven CAD who participated in the Regression Growth Evaluation Statin Study.

Concerning lipid profile, the possibly influence of the R219K polymorphism is still evaluated. For example, Hodoğlugil et al in Turks individuals with low HDL-C concentration found the association of R219K polymorphism with HDL-C concentration [12]. Frikke- Schmidt et al in Danish population did not found any association between R219K polymorphism and individuals with Low or High HDL-C concentration [7]. On the other hand, Kakko et al in Finnish women [13] found the association of 219K allele with higher HDL-C concentration. Opposite, Clee et al [5] reported no differences according to HDL-C concentration between K or R allele, although carriers of K allele had significant lower TGs concentration in relation to carriers of R allele [5]. Furthermore, younger homozygotes of K allele had higher cholesterol efflux and HDL-C concentration compared to homozygotes of R allele. Sandhofer et al [14] studied male population and did not find any association of R219K polymorphism and lipid profile. Also, Cenarro et al [15] evaluated the R219K polymorphism in patients with familial hypercholesterolemia with and without premature CAD and reported that K allele was more frequent in subjects without premature CAD compared to individuals with premature CAD [15]. Li et al [16] investigated the relation of R219K polymorphism with the manifestation of CAD in Chinese patients and did not report any significant correlation. However, the TGs concentration were significant higher and HDL-C concentration significant lower in patients with RR genotype than those with KK genotype. Similarly, Delgado-Lista J et al [17] did not find association of R219K polymorphism and lipid profile.

According to our results from the young Greek female nurses (living and working in the similar conditions) we did not find any association with HDL-C concentration or other lipid parameters. Also, no association between R219K polymorphism and Low or High HDL-C subgroups was found although small number involved in these groups may be a limitation.

### R1587K gene polymorphism

The frequency of R allele of R1587K polymorphism in our study was 68%. Pasdar et al [10] reported 75.9% and Frikke-Schmidt reported 76% [18].

Also, Frikke-Schmidt et al [7] found that this polymorphism is overexpresed in individuals (men and women) with low HDL-C concentration. Furthermore, there was a gradual decrease in HDL-C levels about 0.07 mmol/l (2.7 mg/dl) for RK genotype and 0.11 mmol/l (4.2 mg/dl) for the RR genotype. Tregouet et al [19] stated that R1587K has impact on the apo A1 concentration. Wang et al [20] did not found any relation of R1587K with lipids levels in patients with type 2 diabetes mellitus who were treated with rozigliatoze. The study of Clee et al [5] in Danish population has shown that carriers of K allele had lower HDL-C concentration in comparison with the KK genotype. In a multi-analysis including age, BMI, smoking and TGs as independent variables, the R1587K polymorphism remained significant factor for the prediction of HDL-C concentration. Pasdar et al [10] found the association of R1587K polymorphism and apo A concentration and this was not related to CAD. Cohen et al [21] supported that rare alleles with major phenotypic effects contribute significantly to low HDL-C levels in the general population. However, Tupitsina et al [9] observed that in patients with CAD the R1587K polymorphism did not affect lipids levels. Slatter et al [22] investigated the prevalence of mutations and common SNPs in ABCA1 in 154 low HDL-C individuals and 102 high HDL-C individuals. The R1587K SNP was over represented in low HDL-C individuals. Ksiazek et al in a small study of 50 individuals reported a trend (p = 0.07) in terms of association between TGs concentration and R1587K genotype [23]. In our study no association between R1587K polymorphism and HDL-C levels was found. Also, no association between R1587K polymorphism and Low or High HDL-C subgroups was found. However, individuals with RK genotype had significantly higher TGs, total cholesterol and LDL-C concentration compared to RR genotype.

Reduced circulating HDL-C can be caused by either genetic and/or environmental factors (sedentary lifestyle, diabetes mellitus, smoking, obesity or a diet enriched in carbohydrates). The potential mechanisms of interaction between genetic variations and phenotypes contribution are not fully understood. This happens because each study involved different studying population and the environmental interactions could not be ruled out. Thus, in our study the environmental influence was partially diminished. The advantage of our study was that the study cohort was almost homogenous, since the nurses most of the time were following the same day to day program and were eating in the same school canteen. Thus the influence of diet or physical activities were unlikely, which may partially explain the lack of association of R219K or R1587K polymorphisms and HDL-C concentration. However, the influence of smoking cannot be ruled out since 38.5% of students were smokers.

It is well known from clinical trials, that 1% change in LDL-C is associated with a 1% change of cardiovascular events, which means that individuals born with favorable genotype (in this case RR genotype of R1587K) have already some advantage compare to less favorable genotypes, since they have lower LDL-C. At that time, this was only a clinical observation. Hopefully in near future we will be able to identify high risk patients according to genetic testing, which the assessment in these days has same cost, time implications and replication problems in independent studies, which are disadvantages in routine clinical practice. Nevertheless, these limitations may become less relevant as technology develops.

In summary, the R1587K polymorphism of ABCA1 gene was associated with altered lipid levels in Greek young nurses. Women with RK genotype had higher TGs, total and LDL-C concentration compared to RR genotype. These observations may be significant in assessing the risk of CAD since a 1% change in LDL-C is associated with a 1% change of cardiovascular events and TGs concentration were documented to play a significant role in women.

## Competing interests

The authors declare that they have no competing interests.

## Authors' contributions

VK participated in the development of hypothesis, drafting of the manuscript and carried out the genetic analysis, GK conceived the study and participated in the development of the hypothesis, the study design and drafting of the manuscript, AM participated in the molecular genetic studies, AK performed the statistical analysis and drafting of the manuscript, GV and AK collected the blood samples, DD participated in revising the manuscript critically for important intellectual content, CM and CD participated in the study design and its coordination. All authors read and approved the final manuscript.
